# Exogenous Melatonin Enhances the Cold Tolerance of Harvested Peach (*Prunus persica* Batsch) Fruit by Elevating SAMDC and Decreasing ACC Synthase Activities

**DOI:** 10.3390/plants15162497

**Published:** 2026-08-18

**Authors:** Hongyang Du, Guting Liu, Yuxin Xia, Yiting Guo, Jiabao Tang, Huaipan Liu

**Affiliations:** 1 College of Life Science and Agronomy, Field Observation and Research Station of Green Agriculture in Dancheng County, Zhoukou Normal University, Zhoukou 466001, China; 2 College of Biological Science, China Agricultural University, Beijing 100193, China

**Keywords:** chilling injury, competition, enzyme activity, ethylene biosynthesis, peach (*Prunus persica* Batsch), polyamine biosynthesis

## Abstract

Exogenous melatonin can alleviate cold injury in post-harvested fruit during fruit refrigeration. The mechanism of melatonin-mediated cold tolerance is increasingly being focused on in studies. However, in cold + melatonin-treated fruit, the changes in the activities of *S*-adenosylmethionine decarboxylase (SAMDC) and 1-aminocyclopropane-1-carboxylic acid (ACC) synthase, the key enzymes that catalyze polyamine and ethylene biosynthesis, respectively, are still unclear. In this study, the typical symptoms of cold injury, the contents of polyamines and ethylene, and the activities of SAMDC and ACC synthase are elucidated in melatonin-treated peach fruit under cold conditions. The results show that exogenous melatonin alleviated cold injury in peach fruit and elevated SAMDC activity and, thereby, the levels of spermidine and spermine. More importantly, ACC synthase activity, the ACC level and the ethylene emission rate decreased. These results hint that exogenous melatonin enhanced the tolerance of peach fruit to cold stress by elevating SAMDC and decreasing ACC activities. The results of the inhibitor experiments supplied additional evidence for this hypothesis. The inhibitor methylglyoxal-bis (guanylhydrazone) could inhibit SAMDC activity, decrease the levels of spermidine and spermine, increase the ACC level and ethylene emission rate, and thereby decrease the fruit’s cold tolerance. Meanwhile, the inhibitor aminoethoxyvinylglycine could decrease the ACC synthase activity, the ACC level, and the ethylene emission rate, and also increase the fruit’s cold tolerance. Furthermore, the proposed competitive relationship between SAMDC and ACC synthase for substrate *S*-adenosyl-L-Met is also discussed in this paper.

## 1. Introduction

Post-harvested fruit are often preserved under low-temperature conditions to prolong their shelf life because low temperatures can reduce the metabolic activities of the fruit cells [1,2,3]. Meanwhile, inappropriate treatment with low temperatures can lead to biochemical and physiological disorders and agronomic trait changes in fruit [4], referred to as cold damage. For example, fruit firmness decreases, and browning area, malondialdehyde content and bio-membrane permeability increase [5]. The typical symptoms of cold damage to peach fruit include flesh browning, skin color reddening, increased flesh woolliness and decreased fruit surface firmness [4,5]. Due to this phenomenon, fruit quality deteriorates significantly during post-harvest storage, and storage life is limited. Therefore, determining how to enhance the tolerance of post-harvested fruit to cold stress is increasingly drawing public attention. Treatment with plant growth regulators is one of the most important methods for preserving fruit freshness quality under low-temperature conditions. For example, exogenous polyamine treatment can promote the cold tolerance of carambola [6] and apricot fruit [7]. Min et al. [8] have reported that chilling injury in tomato fruit is alleviated by applying exogenous methyl jasmonate.

It has been reported that melatonin (N-acetyl-5-methoxytryptamine), which is found in mammals and plants, has been supplied to enhance the tolerance of plants to abiotic stresses [9], especially the tolerance of fruit to cold stress, and the research on the mechanism of melatonin-mediated cold tolerance is increasingly interesting [5,10,11]. For example, Cao et al. [5,12] have reported that exogenous melatonin can elevate polyamine levels, enhance oxidation resistance, and thereby alleviate cold damage in fruit. Our previous studies have further shown that melatonin application can maintain bio-membrane conformation stabilization by elevating the levels of conjugated polyamines in bio-membranes [13,14]. Polyamines have been involved in most of these studies. Furthermore, it is well documented that ethylene is involved in fruit ripening, softening and aging [15,16]. However, the relationship between polyamines and ethylene has not been fully elucidated in melatonin-treated fruit under cold conditions.

Polyamines, which are pleiotropic bioorganic plant regulators, are involved in plant growth, development and adaptation to diverse biotic and abiotic stresses [17,18,19,20,21]. Common polyamines include mainly putrescine, spermidine and spermine. Putrescine is mainly biosynthesized from decarboxylation reactions of arginine and ornithine, which are catalyzed by arginine decarboxylase and ornithine decarboxylase, respectively [22,23]. By spermidine synthase catalysis, one amino propyl residue is linked to one end of the putrescine chain to form spermidine and then, by spermine synthase catalysis, another amino propyl residue is linked to the other end of the putrescine chain to form spermine. The donor of the amino propyl residue is decarboxylated *S*-adenosyl-L-Met, which is synthesized from S-adenosyl-L-Met (SAM) by S-adenosyl-L-Met decarboxylase (SAMDC) [24]. Thus, SAM is an important precursor for spermidine/spermine biosynthesis and SAMDC is a key enzyme of the biosynthesis reaction [25], although spermidine and spermine synthases act as important factors in the biosynthesis [26]. It is well documented that spermidine and spermine can enhance the tolerance of plants to many environmental stresses by scavenging directly active oxygen species [20,27,28,29], maintaining function and conformation of organelles and biomacromolecules [13,14] and signaling [20,21,30]. Numerous research works have shown that fruit freshness quality can be improved by increasing SAMDC activity; thereby, shelf life can be extended [31,32,33]. Especially, under cold stress, spermidine and spermine induce defense reactions to alleviate oxidative injury in fruit [5,12] and maintain the structure and function of bio-membranes by being conjugated covalently and non-covalently to bio-membranes of fruit cells [13,14].

Ethylene, which is known for its versatile regulatory functions in plants, is one of the essential phytohormones [34,35,36,37], and many abiotic stresses can promote ethylene emission in most plant tissues [38,39,40,41]. It is, thus, considered as one plant hormone that is involved in senescing, abiotic-stress-induced injury symptom [42] and numerous fruit ripening-related indexes, including firmness, aroma, color, sweetness, etc. [43]. The level of 1-aminocylopropane-1-carboxylic acid (ACC) performs an essential and decisive function in ethylene production [44,45]. It is well known that ACC is synthesized from SAM by ACC synthase. Although ethylene biosynthesis involves many enzymes (e.g., adenosylmethionine synthase and ACC oxidase), which perform irreplaceable roles, ACC synthase is the pivotal enzyme in this process [46,47,48].

As mentioned above, SAM is an important common precursor for both ethylene and spermidine/spermine biosynthesis [23] and polyamine crosstalk with ethylene modulates fruit ripening [16]. However, whether there is a probable competitive relationship between SAMDC and ACC synthase needs to be further elucidated [31,49]. Both ethylene and spermidine/spermine are essential plant growth regulators and perform crucial roles in plant growth and development [16]. Ethylene performs critical roles in maturing, softening and aging of fruit [15,16]. Exogenous polyamine application can lower ethylene release and elevate peach fruit firmness [50]. Inhibiting ACC synthase activity and thereby decreasing ethylene production can delay tomato ripening, improve its freshness quality and extend shelf life [51]. Therefore, to extend fruit shelf life, it is important to prevent fruit from over-ripening, softening and aging. In this sense, it appears that the functions of polyamines and ethylene are antagonistic in alleviating fruit cold injury, which needs to be further fully explored.

Here, exogenous melatonin was supplied to the post-harvested peach fruit during cold storage. We found that melatonin treatment elevated the tolerance of fruit to the cold stress and alleviated cold injury. Furthermore, SAMDC activity and the levels of spermidine and spermine increased in melatonin-treated fruit. Interestingly, the ACC level and the ethylene emission rate decreased markedly with the increase in SAMDC activity. Thus, it could be inferred that exogenous melatonin treatment alleviated cold injury in peach fruit by increasing SAMDC and decreasing ACC synthase activities. This notion was further verified by experiments with inhibitors methylglyoxal-bis (guanylhydrazone) (MGBG) and aminoethoxyvinylglycine (AVG), which can strongly and specifically inhibit the activities of SAMDC [52] and ACC synthase, respectively [43,44].

## 2. Results

### 2.1. Effects of Low Temperature, Melatonin, MGBG and AVG on IPBA, PMRP, Fruit Surface Firmness and Cut Surface Browning Area

**Figure 1:**
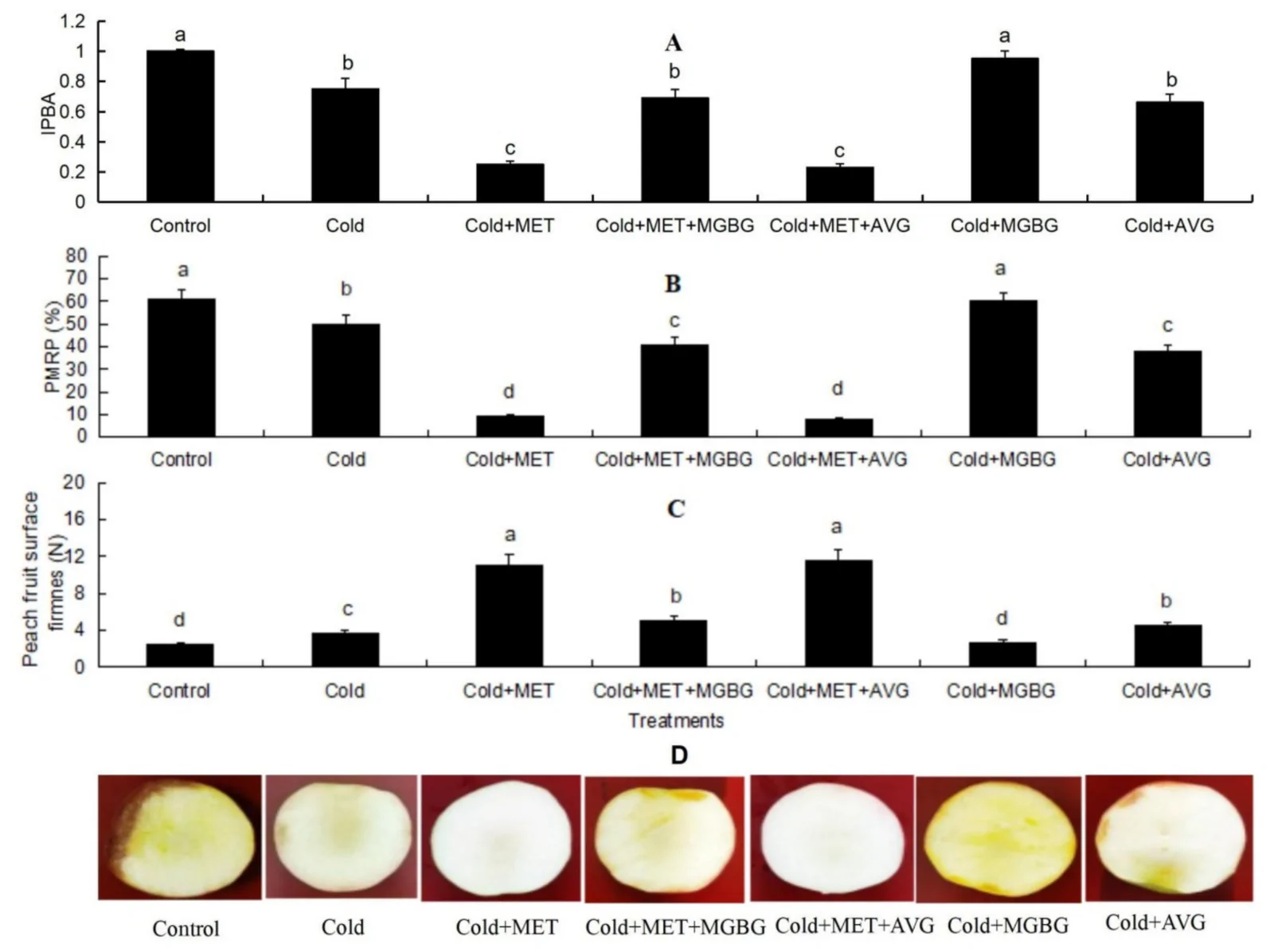
Effects of low temperature, melatonin, MGBG and AVG on IPBA (**A**), PMRP (**B**), fruit surface firmness (**C**) and cut surface browning area (**D**). Control: Peach fruit were immersed into de-ionized water for 30 min. Then, they were air-dried at 25 °C for 45 min. Finally, they were stored for 10 d in the chamber with relative humidity of 80% and room temperature of 25 °C; Cold, Cold + MET, Cold + MET + MGBG, Cold + MET + AVG, Cold + MGBG and Cold + AVG represent the peach fruit of one group treated with de-ionized water, de-ionized water with 0.1 mM melatonin, de-ionized water with 0.1 mM melatonin + 0.2 mM MGBG, de-ionized water with 0.1 mM melatonin + 10 μM AVG, de-ionized water with MGBG (0.2 mM), and de-ionized water with AVG (10 μM), respectively. Then, they were stored for 10 d in the chamber with relative humidity of 80% and low temperature of 4 °C. The significant difference among the multiple averages was analyzed by Tukey’s test (*p* < 0.05) and demonstrated by a different lowercase letter over the column.

Under low-temperature conditions, IPBA (Figure 1A) and PMRP (Figure 1B) decreased (from 1 to 0.75 and from 61% to 50%, respectively); meanwhile, the fruit surface firmness (Figure 1C) increased (from 2.5 N to 3.7 N), compared with the control group of peach fruit stored at 25 °C, indicating that low temperature could extend the shelf life of post-harvested peach fruit. Melatonin treatment increased the fruit surface firmness (from 3.7 N to 11.0 N), and decreased IPBA and PMRP (from 0.75 to 0.25 and from 50% to 9%, respectively) of low-temperature-treated fruit, conveying that melatonin could attenuate cold damage markedly and thereby extend further the shelf life of peach fruit under low-temperature storage. The observation of the peach fruit cut surface (Figure 1D) also verified this notion. From Figure 1, it can be inferred that the effects of melatonin treatment were reversed considerably by MGBG. In cold-treated fruit without melatonin, MGBG application aggravated cold injury in fruit, while AVG mitigated injury, which was judged by cut surface observation and the three indexes mentioned above (Figure 1). The underlying mechanism deserves to be elucidated. In this paper, a probable competitive relationship between SAMDC and ACC synthase, which affected the polyamine levels and ethylene emission rate, respectively, is elucidated as follows.

### 2.2. Effects of Low Temperature, Melatonin, MGBG and AVG on Polyamines in Peach Flesh

**Figure 2:**
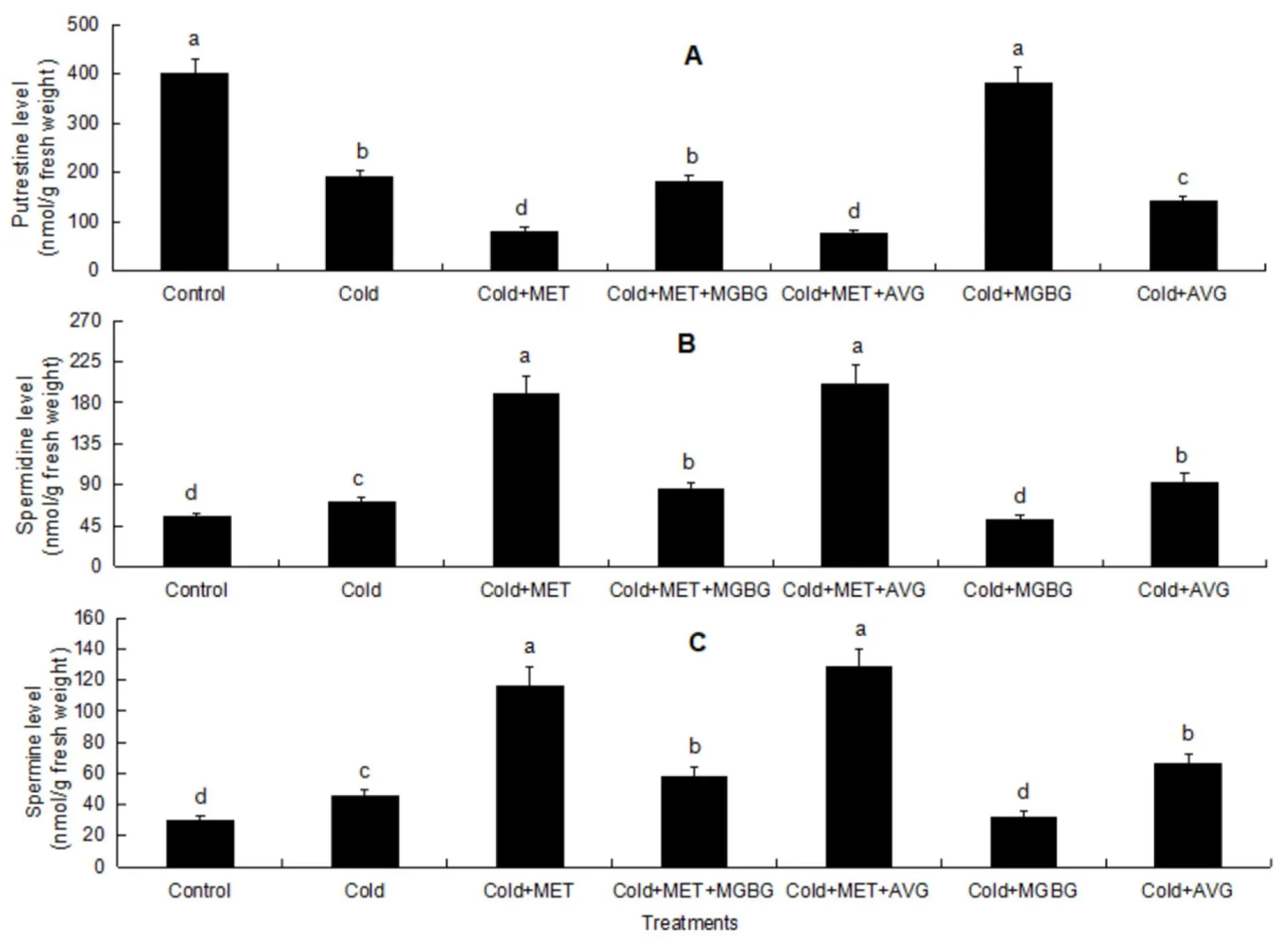
Effects of low temperature, melatonin, MGBG and AVG on polyamines (**A:** putrescine; **B**: spermidine; **C:** spermine) in peach flesh. “Control” and “Cold”, “Cold + MET”, “Cold + MET + MGBG”, “Cold + MET + AVG”, “Cold + MGBG” and “Cold + AVG” mean the same as in Figure 1. The significant difference among the multiple averages was analyzed by Tukey’s test (*p* < 0.05) and demonstrated by a different lowercase letter over the column.

Three main free polyamines in peach flesh, putrescine, spermidine and spermine, were detected in this experiment (Figure 2). Compared to control group fruit, which were stored at room temperature (25 °C), putrescine in low-temperature-treated fruit decreased (from 401 to 190 nmol/g FW) (Figure 2A). It was interesting that melatonin treatment enhanced the decrease in putrescine level (from 190 to 80 nmol/g FW) in peach fruit under low-temperature conditions. More importantly, MGBG reversed the effect of melatonin on the putrescine level substantially, while the effect of melatonin on the putrescine level was not affected by AVG. In cold-treated fruit without melatonin, MGBG also reversed the decrease in putrescine level induced by low temperature from 190 to 380 nmol/g FW, while AVG enhanced the decrease from 190 to 140 nmol/g FW (Figure 2A).

The trends of the changes in spermidine (Figure 2B) and spermine (Figure 2C) were the same under low temperature, melatonin, MGBG and AVG treatments. Compared to the control, both spermidine and spermine increased in the fruit under cold conditions, and melatonin promoted the increases. Interestingly, melatonin-induced increases in spermidine and spermine were markedly repressed by MGBG, while AVG did not affect the increases. In cold-treated fruit without melatonin, MGBG reversed the increases in both spermidine and spermine contents induced by low temperature from 70 to 57 nmol/g FW and from 45 to 32 nmol/g FW, respectively (Figure 2B,C), while AVG enhanced the increases in spermidine and spermine levels from 70 to 92 nmol/g FW and from 45 to 66 nmol/g FW, respectively (Figure 2B,C).

### 2.3. Effects of Low Temperature, Melatonin, MGBG and AVG on the Activities of the Key Enzymes of Polyamine Biosynthesis

**Figure 3:**
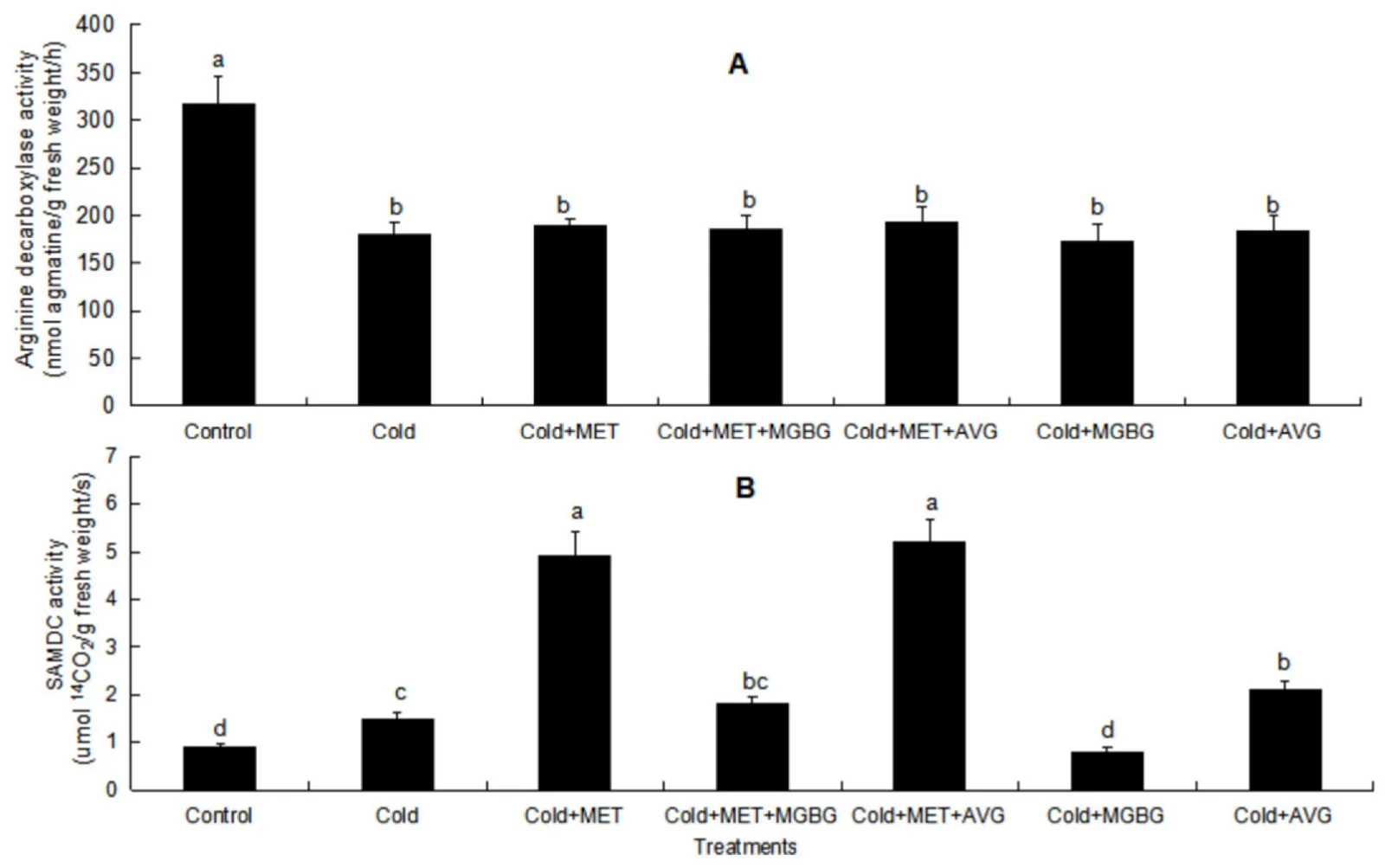
Effects of low temperature, melatonin, MGBG and AVG on the activities of the key enzymes (**A**: Arginine decarboxylase; **B**: SAMDC) of polyamine biosynthesis. “Control” and “Cold”, “Cold + MET”, “Cold + MET + MGBG”, “Cold + MET + AVG”, “Cold + MGBG” and “Cold + AVG” mean the same as in Figure 1. The significant difference among the multiple averages was analyzed by Tukey’s test (*p* < 0.05) and demonstrated by a different lowercase letter over the column.

Arginine decarboxylase is the main enzyme that catalyzes putrescine biosynthesis. Thus, it was necessary to detect arginine decarboxylase activity under low temperature, melatonin, MGBG and AVG treatments. From Figure 3A, it can be seen that arginine decarboxylase activity was the highest in the peach fruit stored at room temperature (25 °C), which was consistent with the result of the putrescine level (Figure 2A). However, low temperature declined arginine decarboxylase activity by nearly half. Furthermore, melatonin, MGBG and AVG treatments slightly inhibited the low-temperature-induced decrease in the enzyme activity, and there was no significant difference among the enzyme activities (Figure 3A). However, from Figure 2A, it can be seen that in the cold + melatonin-, cold + melatonin + AVG-, and cold + AVG-treated fruit, putrescine level was reduced more significantly than that in cold-treated fruit, while in the cold + MGBG-treated fruit, putrescine level increased more markedly than that in the cold-treated fruit. These results suggest that the change in arginine decarboxylase activity was not consistent with the change in putrescine level. This might be attributed to the conversion from putrescine to spermidine and spermine by SAMDC catalysis.

SAMDC catalyzes the rate-limiting step in spermidine and spermine biosynthesis. Therefore, in this paper, it was necessary to detect its activity to further elucidate the relationship between polyamines and ethylene in peach fruit. Figure 3B obviously shows that SAMDC activity increased from 0.9 to 1.5 μmol ^14^CO_2_/g FW/s in the cold-treated peach fruit. More importantly, melatonin treatment enhanced the cold-induced increase in SAMDC activity (from 1.5 to 4.9 μmol ^14^CO_2_/g FW/s). MGBG, an inhibitor of SAMDC, indeed reversed the melatonin effect to a great extent (from 4.9 to 1.8 μmol ^14^CO_2_/g FW/s). However, the melatonin effect was not affected by AVG. Furthermore, MGBG also inhibited the cold-induced increase in the enzyme activity from 1.5 to 0.8 μmol ^14^CO_2_/g FW/s. However, AVG application enhanced the cold- and cold + melatonin-induced increases in SAMDC activity from 1.5 to 2.1 μmol ^14^CO_2_/g FW/s and from 4.9 to 5.3 μmol ^14^CO_2_/g FW/s, respectively (Figure 3B).

### 2.4. Effects of Low Temperature, Melatonin, MGBG and AVG on ACC Content and Ethylene Emission Rate

**Figure 4:**
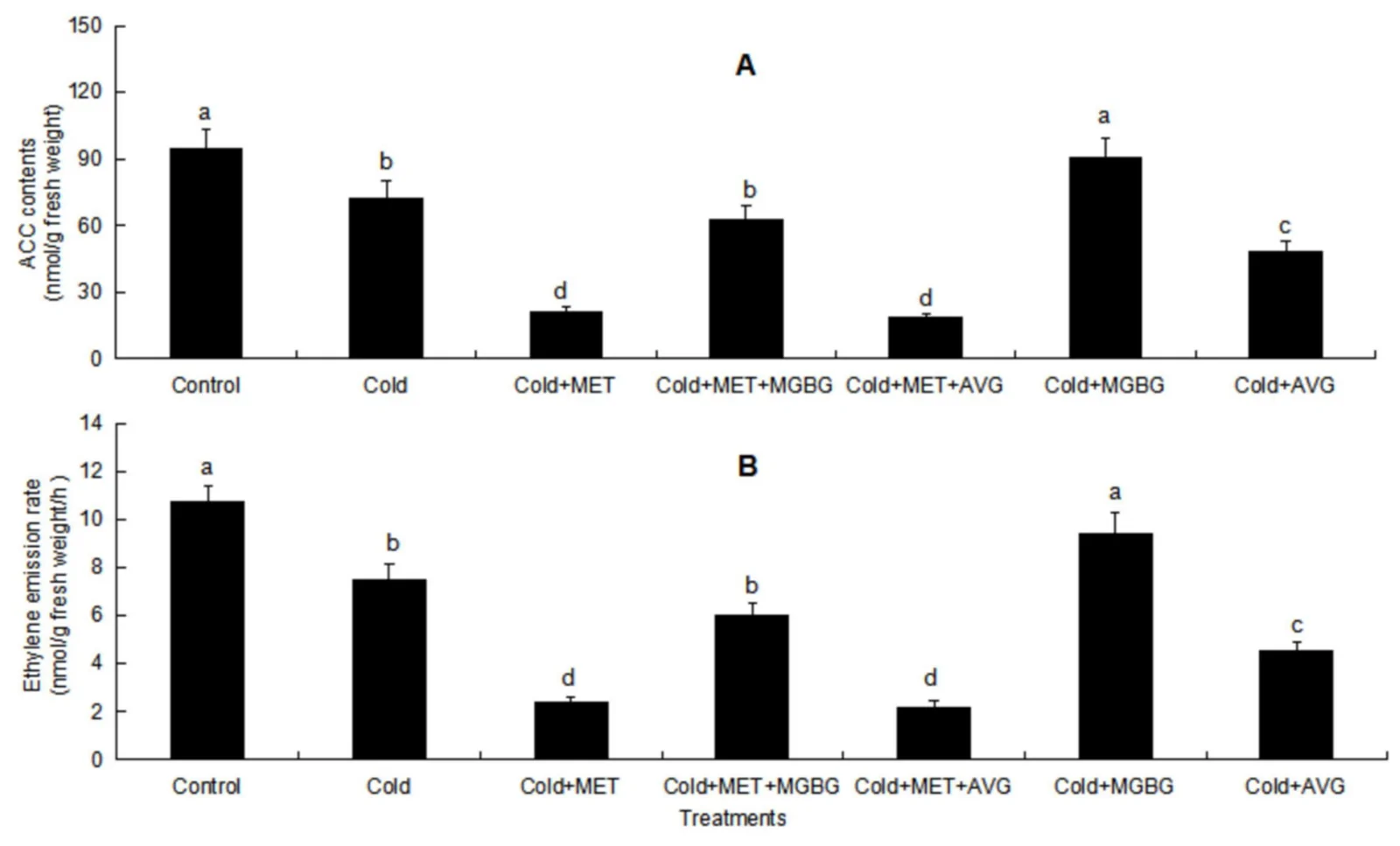
Effects of low temperature, melatonin, MGBG and AVG on ACC content (**A**) and ethylene emission rate (**B**). “Control” and “Cold”, “Cold + MET”, “Cold + MET + MGBG”, “Cold + MET + AVG”, “Cold + MGBG” and “Cold + AVG” mean the same as in Figure 1. The significant difference among the multiple averages was analyzed by Tukey’s test (*p* < 0.05) and demonstrated by a different lowercase letter over the column.

Compared with the control group of peach fruit stored at 25 °C, the ACC content in the fruit stored at low temperature declined from 95 to 72 nmol/g FW, and melatonin treatment markedly facilitated the decrease (from 72 to 21 nmol/g FW) (Figure 4A). MGBG reversed the melatonin effect by 82%, while AVG treatment enhanced the effect by 5.9%. In cold-treated fruit without melatonin, MGBG application elevated the ACC level from 72 to 91 nmol/g FW, while AVG decreased the ACC level from 72 to 48 nmol/g FW (Figure 4A).

The ethylene emission rate followed the same trend of the change as that of the ACC content under low temperature, melatonin, MGBG and AVG treatments. Low temperature decreased the ethylene emission rate (from 10.7 to 7.5 nmol/g FW/h) (Figure 4B), and melatonin application further decreased the ethylene emission rate under cold conditions to a great extent (from 7.5 to 2.4 nmol/g FW/h). However, the effect of melatonin on the ethylene emission rate was markedly reversed by 71% by MGBG. In cold-treated fruit without melatonin, MGBG elevated the ethylene emission rate from 7.5 to 9.4 nmol/g FW/h, while AVG decreased the rate from 7.5 to 4.5 nmol/g FW/h (Figure 4B).

### 2.5. Effects of Low Temperature, Melatonin, MGBG and AVG on ACC Synthase Activity

**Figure 5:**
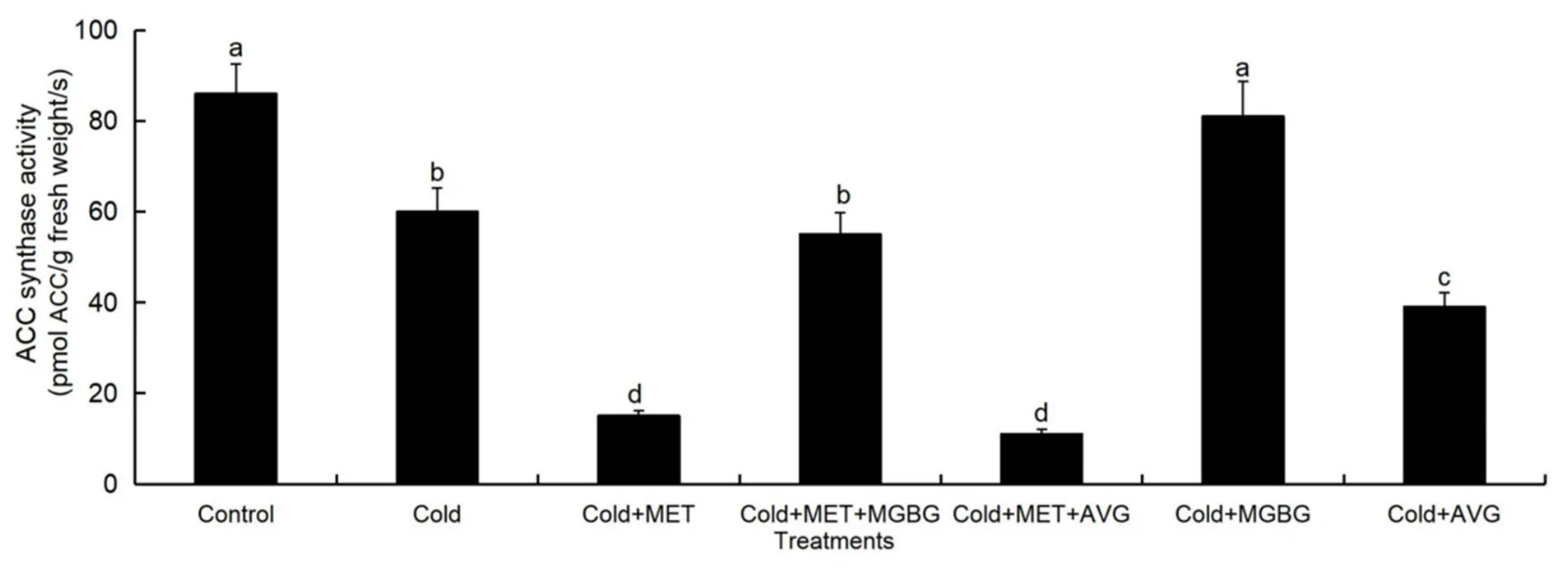
Effects of low temperature, melatonin, MGBG and AVG on ACC synthase activity. “Control” and “Cold”, “Cold + MET”, “Cold + MET + MGBG”, “Cold + MET + AVG”, “Cold + MGBG” and “Cold + AVG” mean the same as in Figure 1. The significant difference among the multiple averages was analyzed by Tukey’s test (*p* < 0.05) and demonstrated by a different lowercase letter over the column.

ACC synthase is the most important enzyme that is closely related to the ACC content and thereby to the ethylene emission rate. Figure 5 shows that ACC synthase activity decreased from 86 to 60 pmol ACC/g FW/s in cold-treated peach fruit. Melatonin treatment notably enhanced the cold-induced decrease in ACC synthase activity from 60 to 15 pmol ACC/g FW/s, and MGBG reversed the melatonin effect considerably from 15 to 55 pmol ACC/g FW/s. The treatment with AVG, an inhibitor of ACC synthase, indeed facilitated the cold + melatonin-induced decrease in the enzyme activity from 15 to 11 pmol ACC/g FW/s. In cold-treated fruit without melatonin, MGBG elevated ACC synthase activity from 60 to 81 pmol ACC/g FW/s, while AVG inhibited the activity from 60 to 39 pmol ACC/g FW/s (Figure 5).

## 3. Discussion

### 3.1. Fruit Low-Temperature Storage, Melatonin and Polyamines

It is well known that low temperature often decreases the activities of the enzymes that are related to cell oxidation. Accordingly, fruit respiratory metabolism is reduced, which results in delaying consumption of fruit nutrients. Thus, low-temperature storage is one of the methods for extending fruit shelf life. However, low temperature often leads to an adverse factor, namely, cold stress. Under cold stress, fruit often exhibits increased IPBA and PMRP, and decreased surface firmness [5,12]. It is currently important to find practicable methods for elevating the fruit’s tolerance to cold stress during low-temperature storage. Treatment with exogenous plant growth regulators is one of the most effective methods for this purpose [6,7,8,9]. As one of the pleiotropic molecules, melatonin can be widely involved in plant growth and development, seed morphogenesis, root architecture, photosynthesis and the resistance of plants to capricious environmental stresses, e.g., cold stress [53]. Therefore, melatonin application is being increasingly paid attention to, and study on the mechanism underlying melatonin-mediated fruit’s cold tolerance is widely interesting [5,10,11]. Most studies have conveyed the information that exogenous melatonin treatment can enhance fruit’s cold tolerance by elevating polyamine levels and thereby scavenging cold-stress-induced active oxygen species [5,12].

In this study, we found that melatonin treatment could alleviate cold damage in peach fruit substantially, which was judged by the decreased IPBA (Figure 1A) and PMRP (Figure 1B), the increased fruit surface firmness (Figure 1C) and the photographs of the fruit’s longitudinal cut surfaces (Figure 1D). Furthermore, in melatonin + cold-treated peach fruit, spermidine and spermine levels increased, while putrescine decreased, as compared with those in cold-treated fruit (Figure 2). These results indicate that putrescine accumulation was disadvantageous to fruit’s cold tolerance, which was consistent with previous studies [13,54]. However, an increasing number of studies have shown that spermidine and spermine are advantageous for plants to improve their resistance to various abiotic stresses [55,56,57,58,59]. In fruit, some studies have indicated that melatonin-induced increases in spermidine and spermine, rather than in putrescine, enhance fruit’s cold tolerance [5,12,13]. In these experiments, under cold conditions, both arginine decarboxylase and putrescine level decreased accordingly. However, in the fruit treated with “Cold + MET”, “Cold + MET + MGBG”, “Cold + MET + AVG”, “Cold + MGBG” and “Cold + AVG”, cold tolerance (Figure 1) and putrescine level (Figure 2A) changed substantially, while the change in arginine decarboxylase activity (Figure 3A) was not consistent with the change in putrescine level. The results conveyed that the conversion of putrescine into spermidine and spermine functioned in enhancing fruit’s cold tolerance, which is in accordance with our previous research [54]. Therefore, SAMDC, the key enzyme that catalyzes the spermidine and spermine biosynthesis from putrescine, should perform a pivotal function in improving fruit’s cold tolerance. SAM is the substrate for an SAMDC-catalyzed decarboxylation reaction. It is known that SAM is also the substrate for an ACC synthase-catalyzed ethylene biosynthesis reaction, and there is an antagonism relationship between polyamine (spermidine or spermine) and ethylene [47]. Is there a competitive relationship between SAMDC and ACC synthase for SAM in cold-treated peach fruit?

### 3.2. SAMDC and ACC Synthase Under Cold Treatment Conditions

Low-temperature treatment leads to a lower ethylene emission rate [48,60] and higher levels of polyamines [5,13]. Here, in cold-treated fruit, coupled with fruit’s cold resistance increasing (Figure 1), SAMDC activity (Figure 3B) and levels of spermidine (Figure 2B) and spermine (Figure 2C) increased accordingly, while ACC synthase activity (Figure 5), the ACC content (Figure 3A) and the ethylene emission rate (Figure 3B) decreased. These results demonstrate that in cold-treated peach fruit, SAM was preferentially used for biosyntheses of spermidine and spermine, rather than for ACC. Previous studies have shown that under normal environmental conditions, SAM in fruit [49,61] and in crop grain [62] is regulated homeostatically, to maintain an appropriate ratio of polyamines (spermidine and spermine) and ethylene production simultaneously, and there is no competitive relationship between polyamines (spermidine and spermine) and ethylene biosyntheses [63]. However, in this study, under cold conditions, the equilibrium of productions of ethylene and polyamines was disrupted by increasing SAMDC activity and there was a competitive relationship between SAMDC and ACC synthase for SAM for fruit to abrogate cold damage. This notion was further verified by experimental results with inhibitors MGBG and AVG, which specifically inhibit SAMDC and ACC synthase, respectively. In cold + MGBG-treated fruit, the activities of SAMDC and arginine decarboxylase (Figure 3) and the levels of spermidine (Figure 2B) and spermine (Figure 2C) decreased, coupled with the decreased fruit’s cold tolerance, accordingly (Figure 1), while ACC synthase activity (Figure 5), the ACC content (Figure 3A) and the ethylene emission rate (Figure 3B) increased. The results imply that with the inhibition of SAMDC by MGBG, adequate SAM substrate was left, which could promote ACC synthase activity by the substrate-induced effect and thereby elevate the ACC content and the ethylene emission rate. Meanwhile, in cold + AVG-treated fruit, ACC synthase activity, the ACC content and the ethylene emission rate decreased, while SAMDC activity and the levels of polyamines (spermidine and spermine) increased, coupled with the increased fruit’s cold tolerance accordingly. The results demonstrate that with the inhibition of ACC synthase by AVG, the substrate SAM could be used by SAMDC for polyamine (spermidine and spermine) biosynthesis.

### 3.3. SAMDC and ACC Synthase Under Cold + Melatonin Treatment Conditions

More importantly, we found that under cold conditions, melatonin treatment further elevated SAMDC activity (Figure 3B) as well as the levels of polyamines (spermidine and spermine) (Figure 2B,C) and peach fruit freshness quality (Figure 1), while melatonin further inhibited ACC synthase (Figure 5) as well as the ACC content (Figure 4A) and the ethylene emission rate (Figure 4B). These findings convey important information: in cold-treated peach fruit, melatonin enhanced the competition of SAMDC with ACC synthase for SAM substrate. This hypothesis was reinforced by the following results with inhibitors. In cold + melatonin-treated fruit, MGBG inhibited SAMDC activity, elevated ACC synthase activity simultaneously and decreased fruit freshness quality considerably. Meanwhile, although the inhibitor AVG markedly inhibited ACC synthase, it did not affect SAMDC activity and fruit freshness quality. These results might be attributed to SAMDC obtaining SAM preferentially in cold + melatonin-treated fruit.

In cold-treated fruit, melatonin elevated the contents of spermine and spermidine by elevating SAMDC activity. Both spermine and spermidine are charged positively and can conjugate non-covalently to bio-macromolecules charged negatively, such as membrane acidic proteins, membrane phospholipids, etc. In addition, spermine and spermidine also can bind covalently to proteins by an amido bond [28,64]. These conjugations can stabilize protein and membrane conformations [13,14]. Therefore, melatonin can attenuate cold damage and elevate fruit freshness quality by elevating SAMDC activity and thereby spermine and spermidine levels. In cold-treated fruit, melatonin inhibited ACC synthase by elevating SAMDC activity and thereby SAMDC competition for SAM, which resulted in decreases in the ACC content and the ethylene emission rate. It is well known that ethylene is closely associated with maturing, softening and aging of fruit [15,16]. Therefore, during fruit cold storage, melatonin can enhance fruit’s cold tolerance and extend shelf life by increasing SAMDC activity and thereby inhibiting ACC synthase activity and the ethylene emission rate. Our findings are supported by Torrigiani et al. [50,65], who have reported that spermidine and AVG can strongly delay peach fruit ripening, extend fresh life and improve fruit freshness quality by impairing ethylene metabolism and signaling, although melatonin was not applied in their experiments.

**Figure 6:**
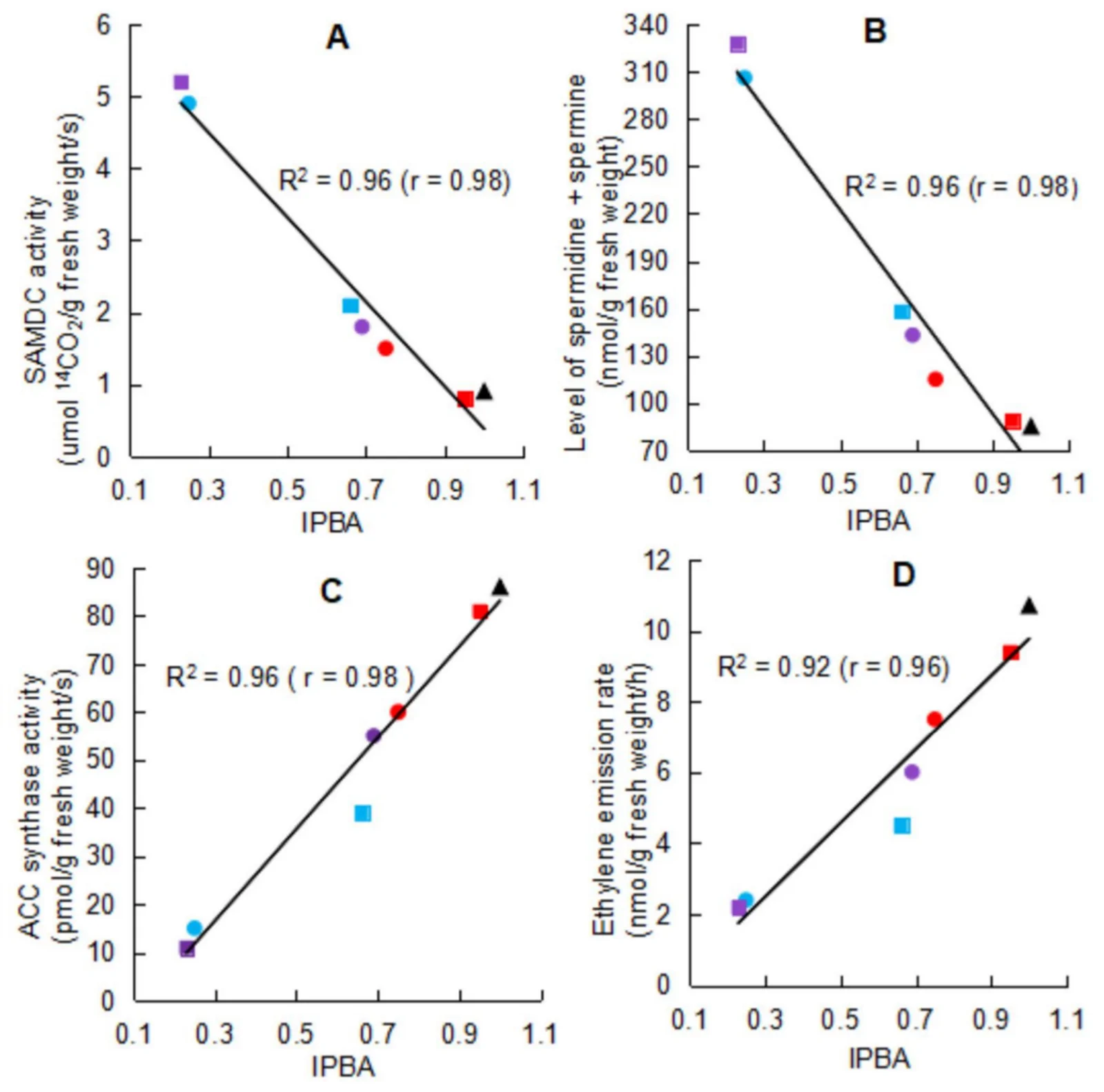
Correlation analysis (**A:** between the fruit cold damage and SAMDC activity; **B:** between the fruit cold damage and the levels of spermidine + spermine; **C**: between the fruit cold damage and ACC synthase activity; **D:** the fruit cold damage and ethylene emission rate). Triangle in black, circle in red, circle in blue, circle in purple, square in purple, square in red, and square in blue represent Control, Cold, Cold + MET, Cold + MET + MGBG, Cold + MET + AVG, Cold + MGBG and Cold + AVG treatment, respectively.

Our viewpoint was further reinforced by the following correlation analysis. Among the symptoms of cold damage to peach fruit, flesh browning was typical in this study. Therefore, IPBA was used for correlation analysis (Figure 6). Obviously, fruit’s cold damage was negatively correlated significantly with SAMDC activity (r_0.05_ = 0.98, n = 7) (Figure 6A) and with the levels of spermidine + spermine (r_0.05_ = 0.98, n = 7) (Figure 6B), while it was positively correlated significantly with ACC synthase activity (r_0.05_ = 0.98, n = 7) (Figure 6C) and with the ethylene emission rate (r_0.05_ = 0.96, n = 7) (Figure 6D). The correlation analysis conveyed that there was an antagonism relationship between SAMDC and ACC synthase. More importantly, from Figure 6, it can be inferred that in cold-treated fruit (circular symbol in blue), melatonin enhanced the competition of SAMDC with ACC synthase for SAM to produce spermidine and spermine; thereby, fruit’s cold tolerance increased substantially. The evidence presented in this paper for the competitive relationship is primarily based on enzyme activity measurements, metabolite levels, inhibitor treatment effects and the above-mentioned correlation analysis. In our future studies, the direct evidence to prove the competitive relationship would be obtained with metabolic flux analysis of SAM, which is labeled with ^14^C isotopes and the gene/protein expression level of SAMDC and ACC synthase.

## 4. Materials and Methods

### 4.1. Harvesting Peach Fruit

The “Yuxian 3” peach (*Prunus persica* Batsch) cultivar, which has been bred by the Agricultural Science Academy in Henan Province of China and is widely planted in North China, was used in this experiment. Although the fruit of this cultivar are big in size and taste delicious, they are cold-sensitive. Thus, to extend their shelf life, it is important to elevate their cold tolerance during low-temperature storage. Previous studies [4,5,12] have shown that peach fruit suffer cold damage under low temperature at 4 °C for 10 d. Our preliminary experiments also showed that storage condition at 4 °C for 10 d could lead to a decrease in surface firmness and increases in browning area and plasma membrane relative permeability. Furthermore, our preliminary experiments indicate that exogenous melatonin at an appropriate concentration (0.1 mM) might alleviate cold injury and extend peach fruit shelf life during cold storage. Additionally, in our preliminary experiments, 0.2 mM MGBG could strongly inhibit SAMDC activity and decrease peach fruit’s cold tolerance, and 10 μM AVG could markedly inhibit ACC synthase activity and decrease the ACC content and the ethylene emission rate. Hence, these concentrations were used to treat the fruit in this study. Under normal horticultural practice conditions, the peach trees were grown in the Zhoukou Horticultural Farm (N: 33°39′/E: 114°38′) in Zhoukou, Henan Province in China. The study was begun in 2023 and peach trees were 6 years old. In June 2023, 2024 and 2025, after fruit growth and nutrient accumulation finished and fruit color and size were fixed, 420 fruits, which were free of disease/pest damage and were uniform in size, shape and color, were picked up. Then in the lab, the fruit were randomly divided into 7 groups with 60 fruit/group and treated as follows.

### 4.2. Treating Peach Fruit

Cold-treated fruit were marked as “Cold”: Firstly, 60 peach fruit of one group were immersed into de-ionized water for 30 min. Then, they were air-dried at 25 °C for 45 min. Finally, they were stored for 10 d in the chamber with relative humidity of 80% and low temperature of 4 °C.

Cold + melatonin-treated fruit were marked as “Cold + MET”: Firstly, 60 peach fruit of one group were immersed into the de-ionized water solution with melatonin (0.1 mM) for 30 min. Subsequently, they were air-dried and cold-stored as in the “Cold” treatment.

Cold + melatonin + MGBG-treated fruit were marked as “Cold + MET + MGBG”: Firstly, 60 peach fruit of one group were immersed into the de-ionized water solution with melatonin (0.1 mM) and MGBG (0.2 mM) for 30 min. Subsequently, they were air-dried and cold-stored as in the “Cold” treatment.

Cold + melatonin + AVG-treated fruit were marked as “Cold + MET + AVG”: Firstly, 60 peach fruit of one group were immersed into the de-ionized water solution with melatonin (0.1 mM) and AVG (10 μM) for 30 min. Subsequently, they were air-dried and cold-stored as in the “Cold” treatment.

Cold + MGBG-treated fruit were marked as “Cold + MGBG”: Firstly, 60 peach fruit of one group were immersed into the de-ionized water solution with MGBG (0.2 mM) for 30 min. Subsequently, they were air-dried and cold-stored as in the “Cold” treatment.

Cold + AVG-treated fruit were marked as “Cold + AVG”: Firstly, 60 peach fruit of one group were immersed into the de-ionized water solution with AVG (10 μM) for 30 min. Subsequently, they were air-dried and cold-stored as in the “Cold” treatment.

The remaining 60 fruit of one group were marked as “Control”: Firstly, 60 peach fruit of the left group were immersed into de-ionized water for 30 min. Then, they were air-dried at 25 °C for 45 min. Finally, they were stored for 10 d in a chamber with relative humidity of 80% and room temperature of 25 °C.

Melatonin, MGBG and AVG were all purchased from Sigma Chemical Co. and their dose concentrations were decided by our preliminary experiments. After treatment for 10 d, fruit were immediately sampled.

### 4.3. The Observation of Peach Fruit Injury

The randomly selected peach fruit from every group were sliced near the stones parallel to the lengthways axis. By the observation of the fruit’s cut surface, cold injury degree could be approximately estimated.

### 4.4. The Evaluation of Peach Fruit Browning Area

In addition to the direct observation of peach fruit injury mentioned above, the degree of cold damage could be further evaluated by comparing fruit cut surface browning area. The index of peach browning area (IPBA) was used and calculated using the method of Du et al. [14] with the following formula:

IPBA = Σ [(fruit flesh browning degree × (fruit number of the relevant browning degree)]/5 × (total investigated fruit number). Here, there were 5 grades by estimating the browning area. Thus, the higher the IPBA was, the more seriously the fruit suffered cold stress.

### 4.5. The Evaluation of Peach Fruit Surface Firmness

A texture analyzer (EZTest/CE-346-51990, Nakagyo, Kyoto, Japan, Shimadzu Co.) was used to detect the firmness of peach fruit surfaces. The penetration force to promote the plunger in fruit could be detected by the analyzer. The peach fruit surface firmness value was the maximum force that promoted the plunger to puncture across the surface, and Newton (N) was used as the unit.

### 4.6. The Evaluation of Plasma Membrane Relative Permeability (PMRP)

PMRP is one of the most important and reliable indexes to indicate injury degree of fruit subjected to abiotic stresses [66]. Here, PMRP was determined using the method of Du et al. [13] with some modifications. Peach flesh tissue (0.5 g) was placed into a test tube with 15 mL de-ionized water. After being shaken in the dark, it was then incubated for 2 h at 25 °C. A portable conductivity meter (DDS-307, Shanghai, China, Shanghai Precision and Scientific Instrument LTD.) was used to measure medium electrical conductivity, which was marked as natural tissue electrical conductivity (NTEC). Then, the flesh tissue was immediately boiled to fully release all electrolytes, and then it was cooled. The medium was measured with the portable conductivity meter and was marked as boiled tissue electrical conductivity (BTEC). DWEC indicated the de-ionized water electrical conductivity. PMRP was calculated using the following formula:

PMRP (%) = (NTEC − DWEC)/(BTEC − DWEC) × 100.

### 4.7. Analysis of the Polyamines in Peach Flesh Tissue

The levels of endogenous free putrescine, spermidine and spermine were detected using the method of Du et al. [13] with some modifications. Peach flesh tissue was homogenized with 5% (*v*/*v*) ice-cold HClO_4_, incubated for 1 h at 5 °C and then centrifuged for 25 min at 15,000× *g*. The polyamines in the supernatant were benzoylated with benzoyl chloride. The benzoylated free polyamines were extracted with cold diethyl ether, centrifuged at 2500× *g* for 10 min, air-dried, re-dissolved with 1 mL methanol (64%, *v*/*v*) and detected using high-pressure liquid chromatography (Waters 2695, Waters Co., Miford, MA, USA). Here, 1 nmol/g fresh weight (FW) was used as one unit of polyamine content.

### 4.8. Analysis of Enzyme Activities of the Key Enzymes of Polyamine Biosynthesis

Arginine decarboxylase activity analysis: Peach flesh tissue was homogenized with the pre-prepared cool extract solution containing phosphate buffer (50 mM, pH 6.3), phenyl methyl sulfonyl fluoride (0.1 μM), ethylene diamine tetraacetic acid (EDTA, 5 mM), phosphopyridoxal (40 μM), vitamin E (20 mM) and dithiothreitol (DTT, 5 mM). The solution was precipitated with (NH_4_)_2_SO_4_ (20–50%), re-dissolved with phosphate buffer (10 mM, pH 6.3), dialyzed for one night and centrifuged at 20,000× *g* for 20 min. Then arginine decarboxylase activity in supernatant was detected using the method of Du et al. [67], and 1 nmol agmatine/g FW/h was used as one activity unit.

SAMDC activity analysis: SAMDC activity was assayed using the method of Du et al. [14] according to released ^14^CO_2_ level, and μmol ^14^CO_2_/g FW/s was used as one activity unit.

### 4.9. Analysis of Ethylene Emission Rate and ACC Content

Ethylene emission rate was detected using the method of Guo et al. [16] with some modifications. Three uniform peach fruit, which were selected randomly, were placed into a 500 mL glass jar, sealed immediately with an airtight stopper and then incubated for 5 h in the dark at 25 °C. A gas sample (1 mL) was withdrawn from the jar headspace with a gastight syringe, and the ethylene emission rate was detected using a gas chromatograph (Solution-2010 series, Shimadzu Co., Kyoto, Japan) with a flame ionization detector and a Porapak Q column (0.18–0.30 mm, 200 cm × 0.3 cm). The temperatures for the detector, column, and injection were invariably kept at 160, 100, and 120 °C, respectively. Here, nmol/g FW/h was used as the unit to express the amount of ethylene released per gram fresh weight of sample per hour.

ACC content was detected using the method of Yang et al. [47] with some modifications. Firstly, in liquid N_2_, peach flesh tissue pieces (50 g) from 3 fruit selected randomly were ground, and powder (0.2 g) was homogenized with 2 mL ethanol (95%) for 10 min at 80 °C. Then, homogenate was centrifuged for 20 min at 3000× *g*, the supernatant was dried under N_2_ and the sample was re-suspended in 2 mL de-ionized water. Chloroform (2 mL) was added into the re-suspended sample to remove lipids. After partitioning, the chloroform fraction was discarded. A sample (0.5 mL) and HgCl_2_ (3.3 mM, 0.2 mL) were placed in a tube. The tube was plugged with a plastic cap and then 0.8 mL NaOCl_2_ (5.5%, *v*/*v*) and 0.4 mL saturated NaOH were injected into the tube. The mixture was vortexed and incubated for 5 min at 27 °C. Finally, the gas sample (1 mL) was withdrawn from the tube with a gastight syringe, and ethylene emission from ACC was assayed using gas chromatography, mentioned above. ACC content was determined by assaying ethylene emission, and 1 nmol/g FW was used as one unit of ACC level.

### 4.10. ACC Synthase Activity Analysis

ACC synthase activity analysis: ACC synthase activity was detected using the method of Woester et al. [68] with some modifications. Peach flesh tissue (50 g) from 3 fruit selected randomly was evacuated for 30 min with a vacuum pump. Then, the sample was ground on ice with a 50 mL solution containing phosphate buffer (250 mM, pH 8.0), EDTA (1 mM), pyridoxal phosphate (10 μM) and DTT (5 mM). Then, the homogenate was centrifuged 2 times for 20 min at 15,000× *g*. ACC synthase was assayed with the final supernatant (5 mL). SAM was added (5 mM, 100 μL) into the supernatant and incubated for 1 h at 30 °C. The reaction was stopped by adding 100 μL HgCl_2_. ACC level was detected using the above-mentioned method, and 1 pmol ACC/g FW/s was used as one ACC synthase activity unit.

### 4.11. Statistical Analysis

The study was carried out three times in three years (2023, 2024 and 2025, once a year). Furthermore, three replicates were designed every year (60 peach fruit of every group mentioned above were further divided into three replicates). Therefore, data here were the average values ± standard error from 9 independent tests and were reliable. Images in Figure 1D are selected as the representatives of 9 independent experimental results of the control and treatments. SPSS 20.0 and Microsoft Excel Software (SPSS Inc., Armonk, New York, USA) were used to analyze data. The significant differences among the multiple averages were analyzed by one-way ANOVA followed by Tukey’s test (*p* < 0.05).

## 5. Conclusions

**Figure 7:**
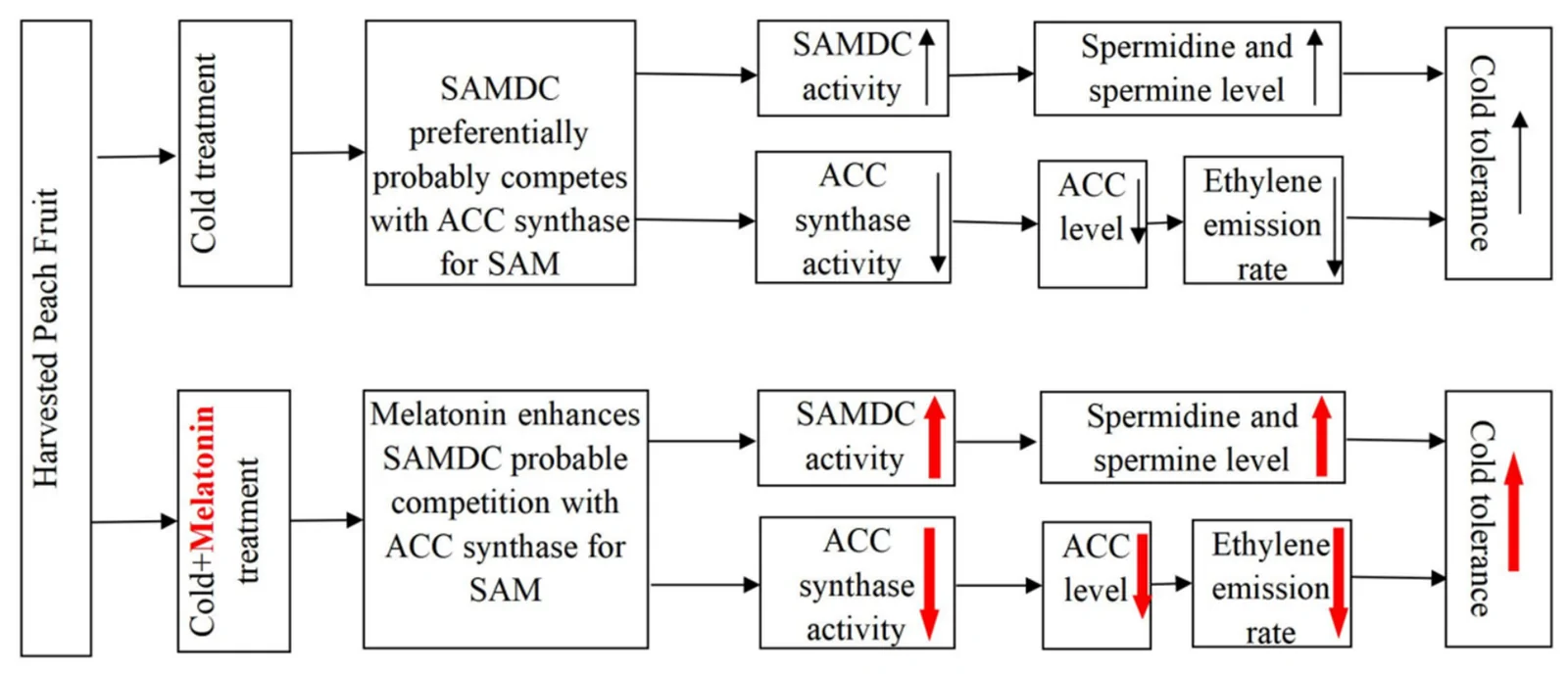
A probable novel mechanism of melatonin-mediated cold tolerance. The horizontal arrow presents the causal relationship. The upwards thin arrow indicates that the trend is increasing, and the red upwards bold arrow indicates that the trend is increasing more markedly. The downwards thin arrow indicates that the trend is decreasing, and the red downwards bold arrow indicates that the trend is decreasing more markedly.

Conclusively, a novel mechanism underlying melatonin-mediated cold tolerance is elucidated here. In cold-treated peach fruit, SAMDC preferentially competes with ACC synthase for SAM, and, more importantly, melatonin application probably enhances the competition, which leads to polyamine (spermidine and spermine) levels increasing and ethylene production decreasing markedly, coupled with the remarkable enhancement of fruit’s cold tolerance. To illuminate the mechanism more clearly, a thumbnail diagram is demonstrated in Figure 7. The finding provides a theoretical basis for melatonin application as an agrochemical to extend shelf life under cold storage. However, on the side of practical application, melatonin of the optimum concentration and the optimum storage temperature should be explored for other, different commercial fruit strains. It is likely that the mechanism underlying melatonin-mediated cold tolerance is considerably complicated and involves phytohormone crosstalk and lots of regulations of related metabolism and gene expression. In the future, with more and more evidence springing up in this area, more insight will become available.

## Data Availability

The original contributions presented in this study are included in the article. Further inquiries can be directed to the corresponding author.

## Abbreviations

ACC	1-aminocyclopropane-1-carboxylate
AVG	Aminoethoxyvinylglycine
DTT	Dithiothreitol
EDTA	Ethylene diamine tetraacetic acid
FW	Fresh weight
IPBA	Index of peach browning area
MGBG	Methylglyoxal-bis (guanylhydrazone)
PMRP	Plasma membrane relative permeability
SAM	S-adenosyl-L-Met
SAMDC	S-adenosylmethionine decarboxylase
TM	Treatment
